# MiR-23-TrxR1 as a novel molecular axis in skeletal muscle differentiation

**DOI:** 10.1038/s41598-017-07575-0

**Published:** 2017-08-03

**Authors:** Neri Mercatelli, Simona Fittipaldi, Elisa De Paola, Ivan Dimauro, Maria Paola Paronetto, Malcolm J. Jackson, Daniela Caporossi

**Affiliations:** 1 Unit of Biology, Genetics and Biochemistry, Department of Movement, Human and Health Sciences, University of Rome “Foro Italico”, Rome, Italy; 2 Laboratory of Cellular and Molecular Neurobiology, CERC, Fondazione Santa Lucia, Rome, Italy; 3 Medical Research Council-Arthritis Research UK Centre for Integrated Research into Musculoskeletal Ageing, Department of Musculoskeletal Biology, Institute of Ageing and Chronic Disease, University of Liverpool, Liverpool, United Kingdom; 4 IRCCS SDN Foundation, Naples, Italy

## Abstract

Thioredoxin reductase 1 (TrxR1) is a selenocysteine-containing protein involved in cellular redox homeostasis which is downregulated in skeletal muscle differentiation. Here we show that TrxR1 decrease occurring during myogenesis is functionally involved in the coordination of this cellular process. Indeed, TrxR1 depletion reduces myoblasts growth by inducing an early myogenesis -related gene expression pattern which includes myogenin and Myf5 up-regulation and Cyclin D1 decrease. On the contrary, the overexpression of TrxR1 during differentiation delays myogenic process, by negatively affecting the expression of Myogenin and MyHC. Moreover, we found that miR-23a and miR-23b - whose expression was increased in the early stage of C2C12 differentiation - are involved in the regulation of TrxR1 expression through their direct binding to the 3′ UTR of TrxR1 mRNA. Interestingly, the forced inhibition of miR-23a and miR-23b during C2C12 differentiation partially rescues TrxR1 levels and delays the expression of myogenic markers, suggesting the involvement of miR-23 in myogenesis via TrxR1 repression. Taken together, our results depict for the first time a novel molecular axis, which functionally acts in skeletal muscle differentiation through the modulation of TrxR1 by miR-23.

## Introduction

Mammalian Thioredoxin Reductase 1 (TrxR1) is a cytosolic selenocystein -containing protein that together with the Thioredoxins (Trx), mitochondrial Thioredoxin Reductase 2 (TrxR2) and NAPDH, constitute the antioxidant Thioredoxin system^1^. By using the NADPH as a source of reducing power, TrxR1 catalyzes the reduction of the active site disulphide in Trx1 which in turn supports the antioxidant cellular response in a variety of different ways^2^. The protective function of Trx-reduced activity includes the peroxiredoxin-dependent reduction of peroxides, the inhibitory effect on ASK-1-dependent apoptosis and the activation of specific redox-sensitive transcription factors as NF-kB, AP-1 and Ref-1^3–7^.

Independently from its antioxidant activity, the modulation of TrxR1 expression has been related to several cellular processes such as stress-induced premature senescence, cell-sufficient growth, embryonic proliferation, DNA replication and malignant transformation^8–11^, thus identifying a role for TrxR1 in cancer progression, and suggesting a putative functional involvement in many other diseases.

TrxR1 expression has been detected in a wide range of different tissues including skeletal muscle, but its function in processes related to muscle biology is still unknown.

Skeletal muscle differentiation is a finely regulated process where mononucleated myoblasts irreversibly withdraw from cell cycle, reach an early step of differentiation (monocytes), and finally fuse into multinucleated myotubes, which are the precursors of mature muscle fibers (terminal differentiation)^12, 13^.

This process is orchestrated by the spatio-temporal-regulated expression and activation of the myogenic regulatory factors (MRFs) including MyoD, Myf5, Myogenin and MRF4, which are specific transcription factors belonging to the basic helix-loop helix family. Together with other general or muscle-specific factors, such as the myocyte enhancer factor 2 (Mef-2), MRFs coordinate muscle development by activating and repressing the expression of a genetic network controlling myogenesis^12^. MyoD and Myf5 are required for the specific commitment to the myogenic lineage, whereas Myogenin is involved at the early step of differentiation^12^.

MicroRNAs are a class of small (21–25 nucleotides) non coding RNAs that regulate gene expression at the post-transcriptional level^14^. Specifically, a single microRNA can inhibit the expression of hundreds of messenger RNAs (mRNAs) by direct pairing between its 5′ end (seed sequence) and complementary sequences on these mRNA targets, usually located in the 3′UTRs^15^. In the last years, a broad number of microRNAs has been found to play a crucial role in skeletal muscle myogenesis^16^. The functional contribution of most of them in regulation of muscle differentiation-related processes has been determined; particularly for the “myomiRs”, a set of microRNAs exclusively or preferentially expressed in striated muscle^16–18^. Beyond myomiRs, other ubiquitously expressed microRNAs appear to be involved in the regulation of muscle development by modulating the expression of several protein targets controlling myogenesis-related cellular processes such as proliferation, migration, epigenetic regulation and cell cycle coordination^16, 19–22^.

miRs-23 family consists of two isomiRs, miR-23a and miR-23b, belonging to the miR-23~27~24 clusters^23^. Because of their ability to regulate proliferation, differentiation and apoptosis in different cell types, the microRNA members of these clusters have been associated with a wide range of human diseases^23, 24^. The expression of these microRNAs has been also detected in skeletal muscle cells where they take part to several muscle-related processes^22, 25, 26^. It has been observed that miR-27b and miR-24 function as positive regulators of skeletal muscle differentiation; in particular, miR-27b ensures the entry of satellite cells into the myogenic program by regulating Pax3 expression^22^, whereas miR-24 promotes terminal differentiation through the TGF-β-mediated pathway^25^. In contrast, the functional role of miR-23a in muscle cells appears related to the protection against skeletal muscle atrophy^26^.

In this paper we describe for the first time that the negative modulation of TrxR1 occurring during skeletal muscle differentiation represents a key step in the establishment of this cellular process, because of its involvement in the transition from proliferating myoblasts to terminally differentiated myotubes. Moreover, we document the regulation of TrxR1 expression by miR-23a and miR-23b through the direct binding to its 3′ UTR. Our findings suggest a novel miRNA-based regulatory network acting on skeletal muscle differentiation.

## Results

### TrxR1 is highly expressed in skeletal muscle tissues and its expression is downregulated during myogenesis

**Figure 1:**
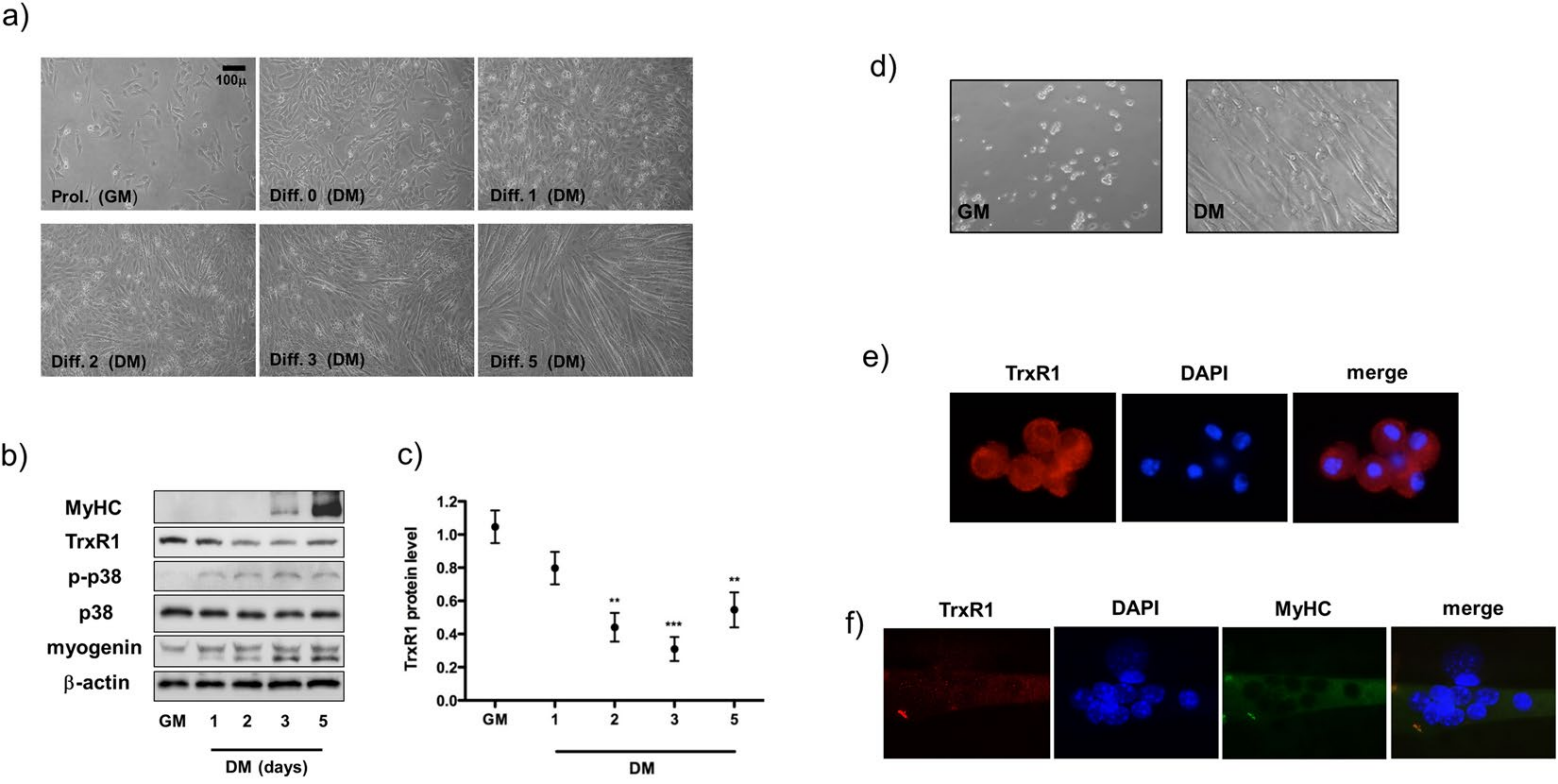
TrxR1 is downregulated during skeletal muscle differentiation. (**a**) Representative image of C2C12 myoblasts differentiation process. Growth medium (GM) is maintained in proliferation conditions (Prol) until 90% confluency. Pictures were taken respectively at 0, 1, 2, 3 and 5 days after switching to differentiation media (DM). (**b**) Western blot analysis showing the TrxR1 protein contents in C2C12 cells cultivated in GM and DM (1, 2, 3, 5 days) conditions. Antibodies against Myogenin, p38, p-p38 and MyHC were used to monitor myogenesis process, whereas β-actin was employed as a housekeeping loading control. (**c**) Relative quantification of TrxR1 protein expression normalized to β-actin levels of western blots in figure b. The TrxR1/β-actin ratio in GM conditions is set = 1. Results are represented as mean ± SEM of three independent experiments. (**d**) Images (10x magnification) of murine satellite cells in proliferation (GM) and 5 days after switching to differentiation media (DM). (**e**) Representative image (63x magnification) of the immunofluorescence analysis showing TrxR1 expression in satellite cells and in (**f**) myotubes originated from them. Multinucleated myotubes are positive for MyHC and DAPI staining was used to detect nuclei.

TrxR1 protein levels were widely detected by western blot analysis in total lysates of several murine skeletal muscle tissues as *gastrocnemius*, *extensor digitorum longus* and *soleus*, as well as in C2C12 cells, a well-established cellular model of murine myoblasts that can recapitulate *in vitro* the process of skeletal muscle differentiation (Supplementary Fig. S1a and Fig. 1a).

In searching for the putative role of TrxR1 in skeletal muscle differentiation, we decided to monitor its expression levels in C2C12 cells under growth conditions and at different days of differentiation ranging from 1 to 5. As shown in Fig. 1b, a marked reduction of TrxR1 protein was observed by western blot analysis during myogenic differentiation of C2C12 cells. Concurrently, myogenic markers of differentiation such as Myogenin, phospho-p38 and MyHC were found to be upregulated, and myotube formation was observed in culture plates between the 3rd and 5th differentiation day (Fig. 1a and b). Down-regulation of TrxR1 protein became significant from day 2 of differentiation (GM *vs* D2 p = 0.0013; GM *vs* D3 p = 0.0005; GM *vs* D5 p = 0.0039) (Fig. 1c). Similar results were obtained by using immunofluorescence analysis. The detection of TrxR1 signal was evident at GM and D0, whereas at D2 and D3 the expression was lower. According to western blot analysis, a recovery of the signal was observed in at least a part of terminally differentiated myotubes (D5) (Supplementary Fig. S1b). A TrxR1 tendency to decrease was observed also in an *ex vivo* model of muscle myogenesis. To this purpose, mouse satellite cells were purified from hind limb muscles and induced to differentiate *in vitro* (Fig. 1d). As shown in Fig. 1e, TrxR1 expression is generally marked in proliferating murine satellite cells (Fig. 1e) and lower in the differentiating myotubes originated from the same cells following *in vitro* differentiation (Fig. 1f). Interestingly the ratio between the oxidative/reduced status of Trx-1, the major target of the TrxR1- mediated antioxidant activity, was unchanged during C2C12 myogenesis, suggesting a Trx-1-independent role of TrxR1 in this specific biological process (Supplementary Fig. S2).

To check if TrxR1 has a functional role in myogenesis, we decided to inhibit its expression in C2C12 cells by an siRNA-mediated strategy and analyse the consequences on differentiation process. As shown in Supplementary Fig. 4a, TrxR1 depletion resulted in a dramatic decrease of C2C12 myogenic rate. Indeed, TrxR1 siRNA transfected myoblasts, submitted to differentiate, exhibited a significant reduction of protein levels of myogenin at D2 (p = 0.031) and MyHC at D3 (p = 0.0012) (Supplementary Fig. 4b). Consequently, this effect resulted in a broad impairment of myotubes formation as monitored by the significant decrease in their fusion index (scr siRNA: 33.2 ± 3.5, TrxR1 siRNA: 8.2 ± 4; p = 0.0075) (Supplementary Fig. 4c and d).

Thus, TrxR1 protein appears to be pivotal in skeletal muscle myogenesis and the fluctuations of its expression levels could have a crucial role in regulating this specific biological process.

### TrxR1 knockdown induces an early myogenesis-related gene expression pattern and reduces C2C12 cell growth, whereas ectopic overexpression delays C2C12 muscle differentiation

**Figure 2:**
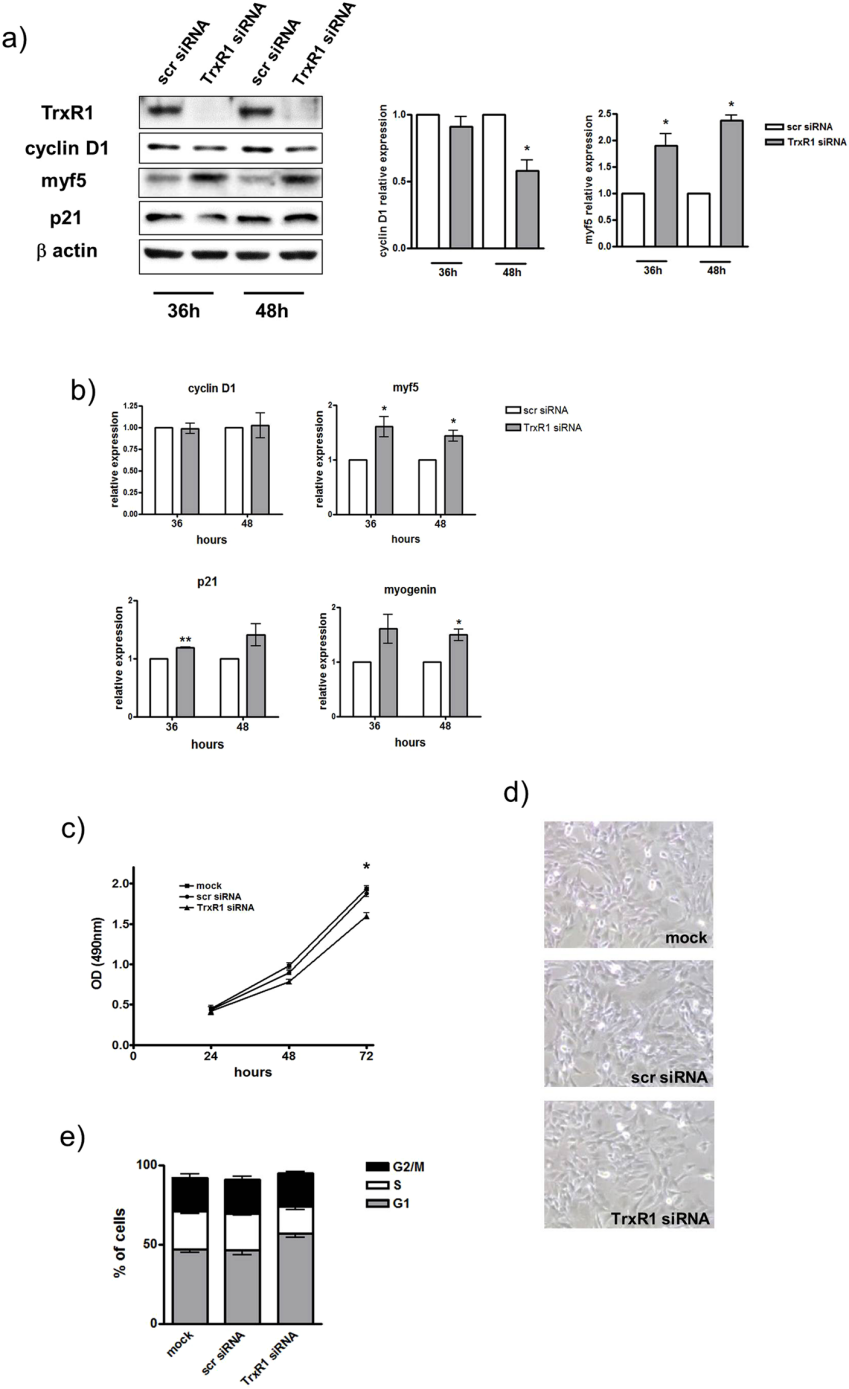
The siRNA-mediated TrxR1 inhibition reduces C2C12 cell growth and induces an early myogenesis-related gene expression pattern. C2C12 myoblasts were transfected with a scramble siRNA (scr siRNA) or a siRNA against TrxR1 sequence (TrxR1 siRNA) and cultivated in GM for 24–72 hours. (**a**) Western blot analysis showing the protein levels of TrxR1, Cyclin D1, p21 and Myf5 at 36 and 48 hours after transfection and the histogram relative to quantification of Cyclin D1 and Myf5 protein levels, represented as mean ± SEM of five different western blot as in panel a. The TrxR1/β-actin ratio of scr siRNA C2C12 is set = 1. (**b**) RT-qPCR analysis showing the mRNA expression levels of Myf5, Cyclin D1, p21 and Myogenin at 36 and 48 hours after transfection. Cyclophilin A mRNA was used to normalize the relative amount of each mRNA analysed. Results are represented as mean ± SEM of at least three different experiments. (**c**) MTS assay measured at 24, 48 and 72 hours after transfection. (**d**) Image (20x) showing cellular confluence at 72 hours. (**e**) Cytofluorimetric analysis showing cell cycle phase distribution.

The differential expression pattern of TrxR1 during muscle myogenesis prompted us to explore its putative functional role in the switch between proliferation and differentiation. Firstly, using an siRNA-mediated strategy we inhibited TrxR1 expression in myoblasts under growth conditions, and then examined the expression of genes related to the early steps of the myogenic program. As shown in western blots in Fig. 2a, 48 hours after siRNA transfection, TrxR1-depleted cells exhibited lower Cyclin D1 and higher Myf5 protein levels with respect to the controls (scr siRNA) (Fig. 2a), whereas no change in p21 was observed (Fig. 2a). In parallel, we performed a RT-qPCR analysis for measuring the expression of Myf5, Cyclin D1, p21 and Myogenin mRNAs in TrxR1 siRNA *versus* control cells. Results obtained showed a significant increase of Myf5 at both time analysed (36 h: 1.6 ± 0.36 fold p = 0.0196; 48 h: 1.44 ± 0.2 fold p = 0.021) and of Myogenin at 48 hours (1.49 ± 0.18 fold p = 0.028) after siRNA TrxR1 transfection (Fig. 2b).

Moreover, p21 mRNA levels were only slightly up-regulated in TrxR1 depleted cells (Fig. 2b), whereas those of Cyclin D1 were unchanged, suggesting, as reported by other groups (Diehl *et al*., 1998, Alteri *et al*., 2013) the putative involvement of a post-transcriptional control taking part in regulation of the protein contents.

Thus, inhibition of the expression of TrxR1 appears to generate a specific gene expression pattern strictly related to that typically shown by C2C12 myoblasts at the early stage of myogenesis, during the transition between their proliferative to terminal differentiation status.

In agreement with this, TrxR1-depleted cells exhibited a reduced growth rate with respect to those observed in scrambled siRNA-transfected and untransfected (mock) cells. Indeed, as shown in Fig. 2d, the OD values measured by MTS assay in TrxR1 siRNA-transfected myoblasts were lower than those of the other experimental groups, reaching a significant decrease at 72 hours (mock: 1.94 ± 0.03, scr siRNA: 1.88 ± 0.044, TrxR1 siRNA: 1.6 ± 0.04; scr siRNA *vs* TrxR1 siRNA p = 0.0033) (Fig. 2c). Concomitantly, a reduced cellular confluence was observed in TrxR1 depleted cells (Fig. 2d). Moreover cell cycle phase distribution analysis showed that myoblasts transfected with TrxR1 siRNA had a significant decrease in S phase population as compared to mock and scr siRNA- transfected cells (mock: 23.6 ± 1.25, scr siRNA: 23.4 ± 1.4, TrxR1 siRNA: 17.3 ± 2.3; scr siRNA *vs* TrxR1 siRNA p = 0,045), with a concomitant increase of the G1 portion (Fig. 2e).

Taken together, these results suggest that TrxR1 decrease during myogenesis may be functionally involved in the coordination of this process, by contributing to the establishment of the genetic network linked to the transition from proliferating myoblasts to terminally differentiated myotubes.

**Figure 3:**
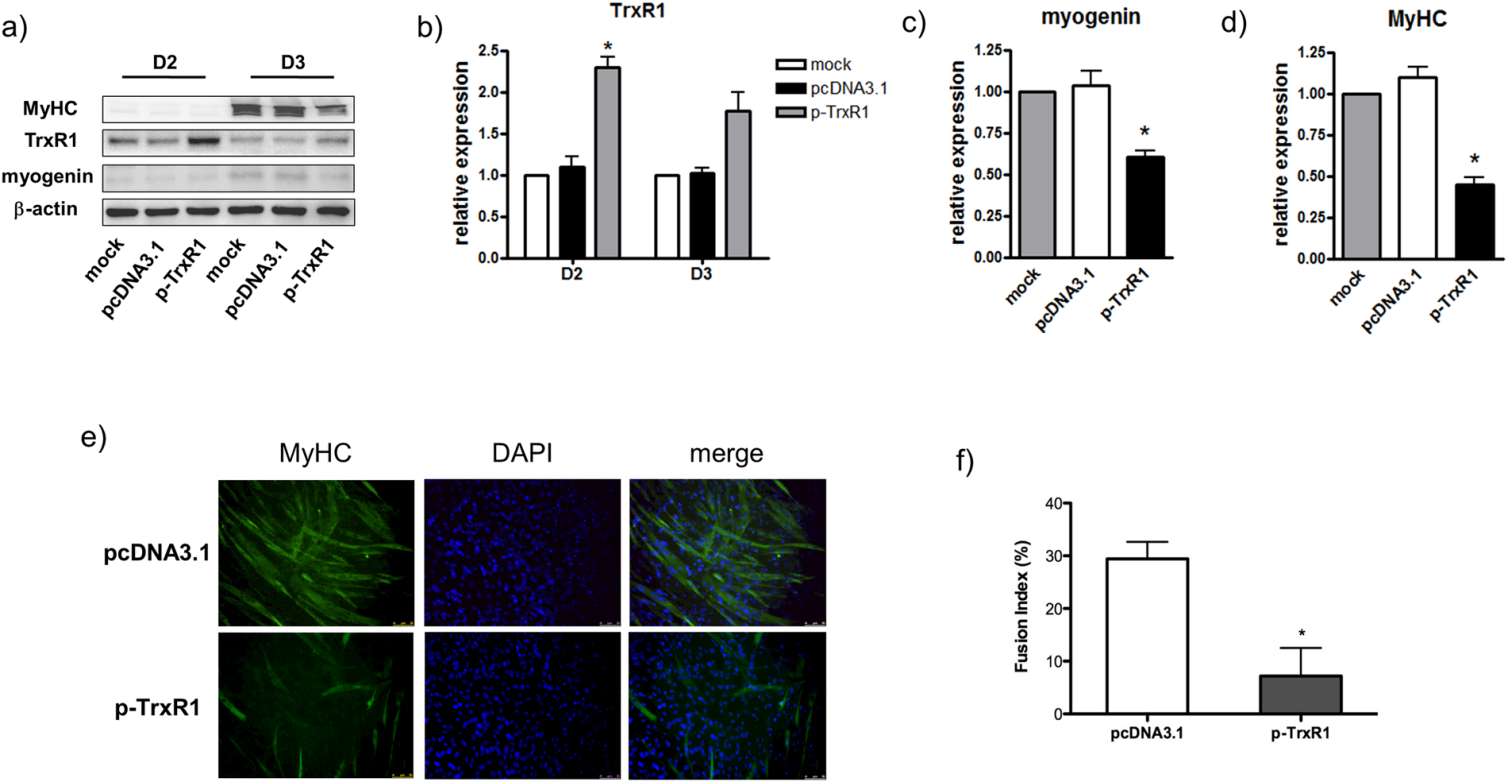
TrxR1 overexpression delays C2C12 differentiation. C2C12 were transfected or not (mock) with p-TrxR1 or the empty vector pcDNA3.1 and analysed at 48 (D2) and 72 hours (D3) from differentiation induction. (**a**) Representative image of the western blot analysis showing the protein expression of TrxR1, Myogenin, MyHC and β-actin. The amount of TrxR1 (**b**), Myogenin (**c**) and MyHC (**d**) in each sample was calculated by normalizing to β-actin protein levels. Results are represented as mean ± SEM of at least three different experiments. The ratio of mock sample was set = 1. (**e**) Representative image (20x magnification) of the immunofluorescence analysis showing MyHC expression at D3 of differentiation. DAPI staining was used to detect nuclei. (**f**) Fusion index relative to D3 calculated in at least five random microscopic fields.

To support this hypothesis, we decided to test whether the rescue of TrxR1 expression levels in C2C12 cells under differentiation conditions could negatively affect their myogenic rate. Therefore, a TrxR1 expression vector (p-TrxR1) or an empty vector (pcDNA3.1) were transfected in C2C12 myoblasts induced to differentiate and the expression of myogenic markers was monitored after 48 (D2) and 72 (D3) hours. As shown in the western blot in Fig. 3a and b, C2C12 cells transduced with p-TrxR1 exhibited an increase of TrxR1 protein levels with respect to control or pcDNA3.1 cultures, ranging from 2.3 to 1.7 fold, at D2 (p = 0.035) and D3 respectively (Fig. 3a and b). The ectopic overexpression of TrxR1 was related to a significant reduction of Myogenin (pcDNA3.1 *vs* p-TrxR1: 1.1 ± 0.1 *vs* 0.6 ± 0.07; p = 0.011) and MyHC expression (pcDNA3.1 *vs* p-TrxR1: 1.1 ± 0.1 *vs* 0.45 ± 0.07; p = 0.0168) measured on the 3rd day of differentiation and/or transfection (Fig. 3c and d). This effect was concomitant with a reduction of myotube formation in TrxR1-transfected cultures as monitored by both, immunofluorescence analysis for MyHC and the related fusion index (p = 0,017) (Fig. 3e and f). These results clearly demonstrate that the rescue of TrxR1 expression levels under differentiation conditions is sufficient to delay the process of C2C12 myogenesis.

### TrxR1 3′UTR harbours two putative binding sites for miR-23a and miR-23b and TrxR1 expression during the early C2C12 differentiation is inversely related to that of both those microRNAs

The differential pattern of TrxR1 expression observed in cycling myoblasts *versus* myotubes prompted us to investigate the molecular regulatory mechanism driving its expression in this specific biological process.

**Figure 4:**
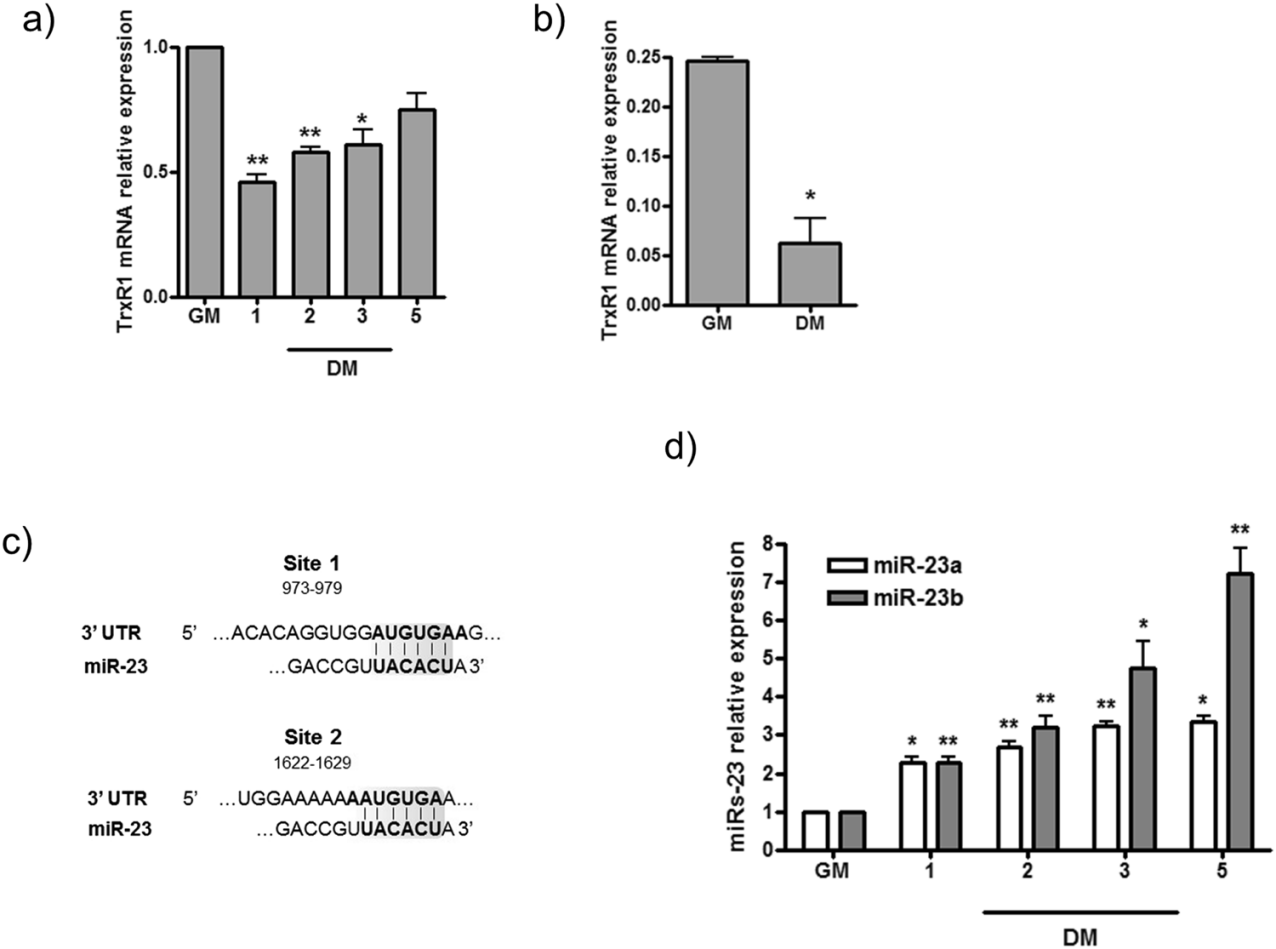
miR-23 is a putative regulator of TrxR1 expression during skeletal muscle differentiation. (**a**) RT-qPCR analysis showing the relative expression of TrxR1 mRNA in proliferating (GM) *versus* differentiating (DM) C2C12 cells collected at 1, 2, 3 and 5 days. Data were normalised to Cyclophilin A expression and represented as mean ± SEM of three independent experiments. (**b**) Relative expression of TrxR1 mRNA in proliferating (GM) *versus* differentiating (DM) satellite cells, measured by RT-qPCR and normalized to GAPDH expression. (**c**) Putative miR-23a and b binding sites in the 3′ UTR of TrxR1 mRNA. (**d**) RT-qPCR analysis displaying miR-23a andmiR-23b expression (normalized to U6 snoRNA) in C2C12 proliferating (GM) versus differentiating (DM) cells. Data are exposed as mean ± SEM of three experiments. The normalised GM values of each miRNA were set = 1.

To do this, we measured the levels of TrxR1 mRNA in C2C12 cells kept in GM or after the switch in DM and collected at different days of differentiation. As shown in Fig. 4a, the results obtained by RT-qPCR clearly showed a 50% decrease of TrxR1 mRNA expression at D1 and D2 and about 40–45% decrease at D3. Interestingly, a smaller reduction was observed in the time period starting from D3, becoming non significant at D5 (Fig. 4a). This specific pattern of expression was also confirmed during the progression into myogenesis of *ex vivo* satellite cells cultivated in proliferative (GM) *versus* differentiation (DM) conditions (Fig. 4b).

One of the mechanisms accounting for the negative regulation of an mRNA levels is that exerted through microRNAs^27^. Given this, we performed a bioinformatic search for putative microRNA binding sites in the 3′ UTR of TrxR1, which is known to be involved in the rapid turnover of TrxR1 mRNA, leading to the deregulation of its protein product^28^.

This analysis, carried out by using *Targetscan* tool (www.targetscan.org), revealed the presence of two putative conserved binding sites for miR-23a and/or miR-23b located at nucleotides 973–979 (site 1) and 1622–1629 (site 2) of TrxR1 3′UTR (Fig. 4c).

To look for a putative relationship linking TrxR1 and miR-23 during myogenesis, we measured the expression levels of both miR-23a and miR-23b in C2C12 myoblasts under GM conditions and at different time points of differentiation. As shown in Fig. 4d, a significant increase of both these microRNAs was observed by comparing GM with DM conditions. In particular, a change ranging from 2.5 to 4.5 fold was measured in the early days of differentiation. Interestingly, on the 5th day of differentiation (D5), miR-23b maintained an increasing trend whereas miR-23a did not (Fig. 4d). Thus, the inverse relationship between the expression of these microRNAs and that of TrxR1 at both protein and mRNA levels observed during early C2C12 myogenesis, suggested us a putative role for miR-23 in the regulation of TrxR1.

### miR-23a and miR-23b negatively affect TrxR1 expression levels by directly binding to its 3′UTR

**Figure 5:**
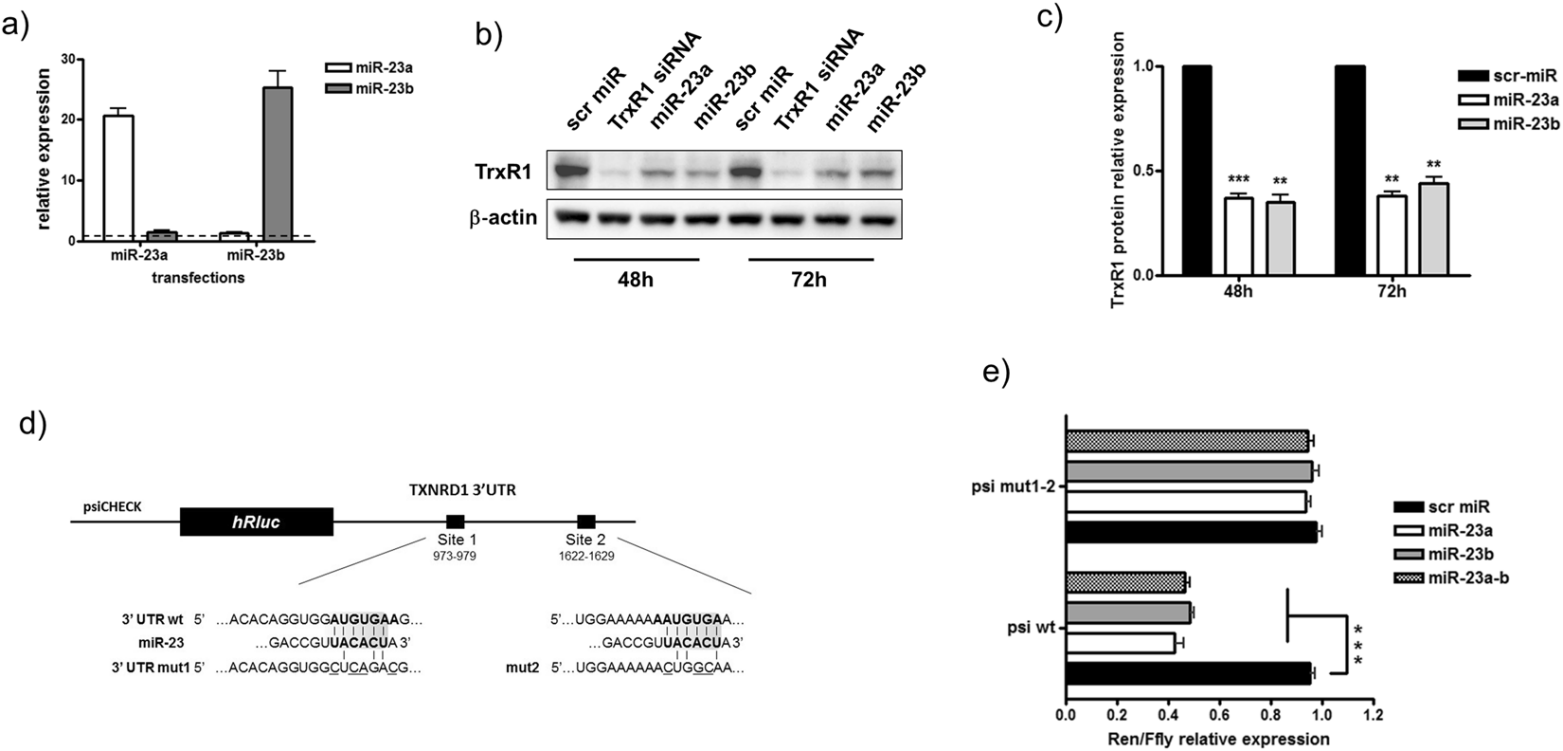
miR-23 overexpression negatively regulates TrxR1 levels by targeting its 3′UTR. C2C12 myoblasts were transfected with mimic miR-23a, mimic miR-23b or a scramble miR (scr miR). (**a**) RT-qPCR showing the expression of miR-23a and miR-23b 48 hours after transfection. (**b**) Representative western blot displaying TrxR1 protein levels at 48 and 72 hours after transfection. The lysates originated by C2C12 transfected with an siRNA against the TrxR1 mRNA sequence (TrxR1 siRNA) were loaded as positive control of TrxR1 downregulation. (**c**) Quantification of panel b performed on three independent experiments. TrxR1 proteins levels were normalized to β-actin and the value of scr miR was arbitrarily set = 1. (**d**) A schematic view of the reporter construct generated by cloning the 3′UTR sequence of TrxR1 mRNA in psicheck vector downstream of renilla luciferase cDNA (psi-wt). Site-specific mutagenesis was performed to generate the reporter construct harbouring the 3′UTR mutated in miR-23 binding sites 1 and 2 (psi mut1-2). (**e**) Histograms showing the relative amount of Renilla/Ffly ratio measured in lysates of C2C12 cells transfected with psi wt, psi mut1 or psi mut1-2 in the presence of scr miR, mimic miR-23a, mimic miR-23b or both (miR-23a-b). The experiment was performed three times. Each biological assay was done in triplicate.

To experimentally validate the putative correlation between miR-23 and TrxR1, we performed experiments to modulate miR-23a and miR-23b in C2C12 myoblasts, and measured the effects on TrxR1 expression levels. We transfected synthetic double-stranded RNA molecules designed to specifically mimic endogenous mature miR-23a or miR-23b. Initially, we monitored by RT-qPCR the efficiency of each one of these molecules in driving the specific over-expression of the corresponding microRNA. Transfections with a synthetic miR-23a increased the specific expression of miR-23a without affecting miR-23b levels. In contrast, C2C12 cells transduced with a synthetic miR-23b displayed high levels of miR-23b but not of miR-23a (Fig. 5a). As shown in Fig. 5b, a robust and significant down-regulation of TrxR1 protein was observed at 48 (miR-23a: p = 0.0009 miR-23b: p = 0.0025) and 72 (miR-23a: p = 0.0012 miR-23b: p = 0.0035) hours after transfections with mimics of miR-23a or miR-23b (Fig. 5b and c). Under the same conditions, the levels of TrxR1 mRNA were found to be negatively modulated by the ectopic expression of these microRNAs (Supplementary Fig. S5a). To explore if the miR-23-mediated downregulation of TrxR1 was able to negatively affect C2C12 growth, we performed an MTS assay in miR-23a, miR-23b and scr miR transfected myoblasts. As shown in Supplementary Fig. S5b, a significant reduction of OD was observed in miR-23a and b-transfected *versus* control (src miR) cells after 72 hours (scr miR *vs* miR-23a p = 0.0062; scr miR *vs* miR-23b p = 0.0094). Thus, the resulting effect was in line with that obtained by the transfection of TrxR1 siRNA (see Fig. 2c), strengthening the hypothesis of the connection between miR-23 and TrxR1 and the consequences in cellular properties due to TrxR1 modulation.

To prove that the observed modulation of TrxR1 was due to a direct action of miR-23 on TrxR1 3′UTR, we generated a reporter construct by cloning the TrxR1 3′UTR downstream of the renilla luciferase reporter gene in psicheck plasmid (psi-wt) (Fig. 5d). Then, a luciferase assay was performed by measuring the renilla/firefly (Luc) activity in C2C12 cells transfected with psi-wt together with either a scrambled microRNA (scr miR) or mimic miR-23a, miR-23b or miR-23a plus miR-23b (miR-23a-b) (Fig. 5e).

As shown in Fig. 5e, transfections with either miR-23a or miR-23b alone, or miR-23a-b, were all able to induce more than a 2 fold decrease of luciferase activity levels with respect to those observed in scr miR transfections (Fig. 5e).

The same experiment was also performed by transfecting cells with a construct harbouring a 3′UTR mutated in miRNA-23 binding sites (psi mut1-2) (Fig. 5d). The effect of miR-23a, miR-23b and miR-23a-b mimics on luciferase activity was completely abolished in cells transduced with psi mut 1-2, suggesting a direct role of these sites in mediating the miR-23- dependent TrxR1 regulation (Fig. 5e).

### LNA-mediated inhibition of miRs-23 counteracts the TrxR1 downregulation in C2C12 differentiation and negatively affects Myogenin and MyHC expression

To identify a functional role in the process of muscle differentiation to the molecular axis linking miR-23 to TrxR1, we decided to transfect C2C12 myoblasts with a specific antisense inhibitor (LNA) of both miR-23a and miR-23b, and determine whether this would prevent TrxR1 downregulation during differentiation as well as in counteracting myogenic program.

**Figure 6:**
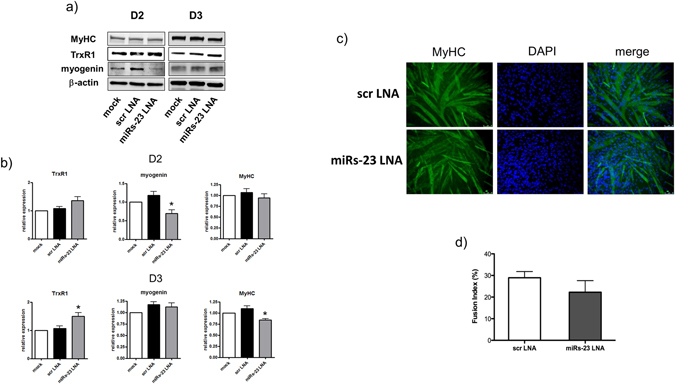
miRs-23 LNA transfection negatively affects Myogenin and MyHC expression and counteracts the downregulation of TrxR1 during C2C12 differentiation. C2C12 were transfected or not (mock) with miRs-23 LNA or a scramble LNA (scr LNA) and analysed at 48 (D2) and 72 hours (D3) from differentiation induction. (**a**) Representative image of the western blot analysis showing the protein expression of TrxR1, MyHC, Myogenin and β-actin. (**b**) The amount of TrxR1, MyHC and Myogenin in each sample was calculated by normalizing to β-actin protein levels. Results are represented as mean ± SEM of at least three different experiments. The ratio of mock sample was set = 1. (**c**) Representative image (20x magnification) of the immunofluorescence analysis showing MyHC expression at D3 of differentiation and the related fusion index (**d**). DAPI staining was used to detect nuclei.

As shown in Fig. 6a, myoblasts transduced with miRs-23 LNA exhibited higher levels of TrxR1 protein than control cells transfected with a scramble LNA. This effect was particularly evident on the 3rd day of differentiation (D3) as shown in Fig. 6b (scr LNA: 1.06 ± 0.09 *vs* miRs-23 LNA: 1.5 ± 0.12; p = 0.048). In order to understand if the resulted up-regulation of TrxR1 by miRs-23 LNA was effective in delaying C2C12 myogenesis, we monitored the expression of Myogenin and MyHC as markers of terminal differentiation. As expected, western blot analysis performed at D2 and D3, revealed a slight but reproducible downregulation of Myogenin at D2 (scr LNA: 1.18 ± 0.1 *vs* miRs-23 LNA: 0.7 ± 0.09; p = 0.014 (Fig. 6a and b) and MyHC at D3 (scr LNA: 1.1 ± 0.06 *vs* miRs-23 LNA: 0.84 ± 0.03; p = 0.016) (Fig. 6a and b) in cell cultures transfected with miRs-23 LNA.

These results prompted us to investigate about a chance of delayed myogenesis due to the observed downregulation of myogenin and MyHC. The fusion index measured in myotubes cultures with scr LNA and miRs-23 LNA (Fig. 6c) did not show any significant change, although a tendency to decrease in miRs-23 LNA was appreciated (Fig. 6d).

Taken together, these results support a role of miR-23-dependent downregulation of TrxR1 in the regulation of myogenic markers and its putative involvement in the process of skeletal muscle differentiation.

## Discussion

Although a series of findings suggest a putative role of TrxR1 in muscle myogenesis, its functional involvement in this cellular process has never been addressed until now. In particular, it is intriguing that TrxR1 expression levels are down-regulated during the *in vitro* differentiation of muscle myoblasts as well as of satellite cells^29, 30^ (see Fig. 1).

In this manuscript, we describe for the first time that the antioxidant enzyme TrxR1 plays a functional role in skeletal myogenesis, and we identify a miR-23-dependent post-transcriptional regulatory mechanism acting on muscle differentiation through the direct modulation of TrxR1 expression levels.

Our findings indicate that the reduced expression of TrxR1 observed during the differentiation of C2C12 myoblasts and mouse satellite cells seems to represent a key step in the this finely regulated cellular process. To prove this, we undertook a series of experiments demonstrating that: a) inhibition of TrxR1 in myoblasts generates a pro-differentiation stimuli (Fig. 2), and b) overexpression of TrxR1 is able to delay C2C12 differentiation (Fig. 3). In myogenesis, the processes of proliferation and terminal differentiation are mutually exclusive and it is well known that these are finely coordinated by molecules controlling cell cycle exit as well as induction of MRFs^31^. Consistent with this, we found that TrxR1-depleted cycling myoblasts show a reduction of Cyclin D1 protein levels, a well-known inhibitor of muscle myogenesis^32^ and a concomitant enhancement of Myf5. As reported by Wang and colleagues, Myf5 is up-regulated at the early stage of C2C12 differentiation and its forced repression negatively affects the myogenic rate of these cells^33^. Additionally, we also observed an increase in Myogenin mRNA levels which is recognized as one of the master regulators of myogenesis, activated in early differentiation^12^. Thus, the downregulation of TrxR1 could be crucial in the establishment of such a gene expression pattern promoting the withdrawal from cell cycle and the entry of myoblasts into the myogenic process. Moreover, this molecular event appears to be necessary for efficient muscle differentiation, because, as clearly shown in Fig. 3, a forced increase of TrxR1 protein levels in myoblasts, strongly reduces their myogenic ability. Collectively our data underline the functional relevance of the switch in TrxR1 content in the differentiation process. Indeed, even the disruption of this physiological expression pattern by siRNA interference during the early phases of differentiation, strongly impair C2C12 myogenesis (see Supplementary Fig. 4). Thus, the establishment of such expression pattern seems to be critical for the right execution of this biological process. Accordingly, it could be reasonable to hypothesized that only TrxR1 highly-expressive myoblasts or myocytes undergo myogenesis through a fine and temporal mechanism of TrxR1 negative modulation, suggesting the pivotal role of this protein and its fluctuations in muscle development.

The mechanism of action by which TrxR1 exerts its role in skeletal muscle differentiation is still poorly understood. To look for a putative involvement of TrxR1 in the ASK-1 dependent-p-38 MAPK activation by its main target, Trx1, we attempted to correlate the physiological TrxR1 downregulation to the oxidation of Trx1, but we did not observe any alteration of the Trx1 oxidative status in C2C12 during differentiation (Supplementary Fig. S2). Thus, as already proposed by Arner and colleagues^34^ TrxR1 may exert its function in a Trx1 -independent manner. Moreover, we may even speculate whether this is a redox-independent role, a conclusion that may be supported by the evidence linking the redox function of the antioxidant enzymes in muscle differentiation to the overall tendency in increasing their expression levels^30, 35^.

However, our main findings highlight the functional importance of a decrease of TrxR1 in skeletal muscle differentiation.

In this context, the identification of miR-23 as a new molecular regulator of TrxR1 expression and its role during skeletal muscle differentiation is of great importance. In agreement with microRNA biology, we found that the regulation of TrxR1 expression is mediated by the direct binding of miR-23a and miR-23b on its 3′UTR. Results obtained by our experimental approach (i.e. overexpression of miRNA mimics and evaluation of their effect on TrxR1 expression levels and 3′UTR activity of Fig. 5) clearly validate this molecular relationship and point to a post-transcriptional regulatory mechanism acting on TrxR1 3′ UTR. In agreement with this, published studies have described the pivotal role of TrxR1 3′ UTR in the regulation of this protein^36^. In particular there are reports that 3′ UTR of TrxR1 contains several AUUUA motifs (AU-rich-elements), also recognized as AREs, which are functionally involved in its mRNA turnover^28, 36^. Moreover, our *in silico* analysis revealed that a putative consensus sequence for the binding of CPEB (cytoplasmic polyadenylation element binding protein) is embedded in this 3′ UTR (data not shown). Thus, the newly identified miR-23 mediated regulatory mechanism is likely to take part, with other classes of molecules (i.e CPEB, or AREs binding proteins) in the complex post-transcriptional regulation acting on TrxR1 expression via its 3′UTR. Moreover, given the relevance of TrxR1 in several human diseases including cancer^37, 38^, the identification of a novel mechanism which can modulate its expression may open new interesting perspectives.

The molecular axis linking miR-23 to TrxR1 appears to be active in the early phases of myogenic process. Indeed, the inverse correlation between the expression of these miRs and TrxR1 is maintained up to D2 of differentiation. By D3 to D5 the displayed pattern appears to be incongruent: miR-23a remains unchanged, miR-23b undergoes a strong increase and TrxR1 tends to rise, suggesting a loss of the regulatory circuitry. This specific readjustment could reflect the well known peculiarity of miR-23a and b in differentially changing their pattern of expression as well as their target activity^39^, thus suggesting divergent role at the late differentiation. However, the, role of this molecular axis in myogenesis, firstly suggested by the inverse correlation between the expression levels of miR-23a and miR-23b and those of TrxR1 observed during C2C12 differentiation, is supported by results obtained from experiments on the specific inhibition of miR-23. As shown in Fig. 6, the rescue of the TrxR1 levels in myoblasts transfected with a miRs-23 LNA and switched to differentiation conditions, correlates to a slight but reproducible reduction of myogenic markers, thus suggesting the involvement of miR-23 in that process via TrxR1 modulation, and consequently strengthening the role of TrxR1 as negative regulator of muscle differentiation. Indeed, even a partial rescue of TrxR1 levels during differentiation is able to affect both Myogenin and MyHC proteins Probably because of their slight modulation, the consequent effect on muscle differentiation seems to be not morphologically relevant although a reduced trend in the fusion index was observed. Interestingly, the failure of a full restauration of TrxR1 levels could suggest the putative involvement of other regulatory mechanisms acting together with miR-23. Moreover, we cannot exclude methodological limits, as for example those due to the transient transfection, that probably does not provide a high transduction efficiency.

The known miR-23 biology appears support our findings in multiple ways. As reported by Wada and colleagues, miR-23 sustains skeletal muscle hypertrophy through downregulation of MAFbx/Atrogen1^26^, and blocks cell cycle progression in endothelial cells^40^. In addition a series of enzymes such as Peroxiredoxin 3^41^ and mitochondrial glutaminase^42^ associated, like TrxR1, to redox homeostasis, have been shown to be direct targets of miR-23 in non-muscle cells.

Thus, our results extend those findings by including miR-23a and miR-23b in the wide class of muscle differentiation noncoding RNAs, where even miR-27 and miR-24 which are clustered together with miR-23, take part. As already reported, the coordinated association of microRNAs from the same cluster toward a specific function is challenging^23, 43^ and, in this specific context, could prove to be effective in enhancing myogenesis functionality.

In conclusion, we have identified a novel molecular axis which functionally acts in skeletal muscle myogenesis through the modulation of the antioxidant enzyme TrxR1 by miR-23. These results might be useful in the field of muscular diseases or in other TrxR1-linked pathological conditions such as carcinogenesis.

## Methods

### Cell preparation and FACS isolation

Hind limb muscles from C57/BL6 wild type mice (Charles River Laboratories) were minced and digested in PBS (Sigma) containing 0.1% BSA, 300 µg/ml Collagenase A (Roche), 0.24 U/ml Dispase I (Roche), 2 μg/ml DNase I (Roche), 50 μM CaCl2 and 1 mM MgCl2 for 60 min at 37 °C under constant agitation. For fluorescence-activated cell sorting, digested muscle cells were stained with primary antibodies (1:50) CD3-eFluor450 (eBioscience), CD45-eFluor450 (eBioscience), Ter119-eFluor450 (eBioscience), Sca-1-FITC (BD Pharmingen), and α7integrin-APC (AbLab) for 30 min at RT. Cells were finally washed and resuspended in Running Buffer (PBS, 0.1% Sodium Azide, 0.2% FBS). Flow cytometry analysis and cell sorting were performed on a DAKO-Cytomation MoFlo High Speed Sorter.

Muscle satellite cells (SCs) were isolated as Ter119−/CD45−/CD31−/α7-integrin+/Sca-1− cells. Fibro-adipogenic progenitors (FAPs cells) were isolated as Ter119−/CD45−/CD31−/α7-integrin−/Sca-1+ cells, as previously described^44^.

### Cell cultures

C2C12 mouse myoblasts were cultivated in DMEM/high glucose supplemented with FBS (10–15%) (growth media; GM) as previously described^45^. To induce myogenic differentiation, cells reaching a confluence of about 75–80% were switched in FBS-low (2%) DMEM media (Differentiation media; DM) and collected and/or analyzed after 24 (D1), 48 (D2), 72 (D3) and 120 (D5) hours.

1 × 10^4^ freshly sorted SCs were plated on the 0.1% gelatin-coated bottom of a 24-well plate, while 0.5 × 10^4^ FAPs cells were plated on the upper insert. Transwell co-cultures were maintained in growth medium (GM) (DMEM GIBCO containing 20% FBS, 10% horse serum and 1% chicken embryo extract) for 7 days and then harvested for analyses (Immunofluorescence or qPCR) or induced to differentiate. For differentiation, SCs were incubated for 7 days in differentiation medium (DM) (DMEM (Gibco) containing 2% horse serum, 0.5% chicken embryo extract.

### Transfections

Plasmid transfections of C2C12 cells were directly performed in DM by using Lipofectamine 3000 Reagent (ThermoFisher Scientific) following manufacturer’s instructions.

Lipofectamine RNAiMAX transfection reagent (ThermoFisher Scientific) was used for short RNA molecule (siRNAs, mimic miRNAs, anti miRNAs, LNAs) transfections. In protocols of myoblast growth, 5500 cells/cm^2^ were reverse-transfected with 20 nM of short RNA molecules and collected at different time points (from 24 up to 72 hours) for further analysis. In differentiation protocols, 2 × 10^5^ cells were reverse-transfected with 60 nM of LNAs or siRNAs and the day after media was replaced by DM.

Anti-miR-23a-3p (AM10644), anti-miR-23b-3p (AM10711), mirvana miRna mimic hsa-miR-23a-3p (MC10644), mirvana miRna mimic has-miR-23b-3p (MC10711), silencer pre-designed siRNA anti Txnrd1 (184264), silencer negative control siRNA (AM4611) were purchased from Ambion (Life technologies) and used at a final concentration ranging from 10 to 25 nM. Has-miR-23 mircury LNA microRNA family inhibitor (450025) was purchased from Exiqon.

Three different siRNAs targeting TrxR1 in C2C12 were assayed. A similar effect on myf5 and pax7 gene expression was observed by comparing siRNA N°1 with N°2, although the siRNA N°1 displayed a higher efficiency in depleting TrxR1 expression (Supplementary Fig. 3a and b). Then we decided to choose the siRNA N°1 from now, on named TrxR1 siRNA, in the following experiments.

### Plasmid constructs

The mouse cDNA sequence of thioredoxin reductase 1 (cytoplasmic isoform 2) open reading frame was amplified by PCR using the following primers: Fw sense-5′aatgcggccgccaaaagctgccaacaatgaa-3′ and Rv antisense-5′cagctcgaggccttcctgaacttcacctg-3′. The amplified product was cloned into the NotI and XhoI sites of pcDNA3.1(+) vector to generate the p-TrxR1 plasmid.

The TrxR1 3′UTR sequence was amplified by PCR from human genomic DNA using the primer Fw sense-5′cagctcgaggttaagccccagtgtggatg-3′ and Rv antisense 5′aatgcggccgcagtaagaaggcacacgtgg-3′ and cloned into XhoI and NotI sites of psicheck^TM^-2 vector (Promega) (psi-wt).

The mutated versions of psi-wt in miR-23 binding sites were obtained by Site-directed mutagenesis. For psi mut1 generation, psi wt plasmid was amplified by a couple of primers carrying the miR-23 mutation in site 1 (Fw-sense 5′cacaggtggacggcaaggattttcatttaaaaacc-3′, Rv-antisense 5′ggtttttaaatgaaaatccttgccgtccacctgtg-3′). After DpnI (New England Biolabs) digestion, the newly synthetized plasmid was transformed into competent *E*. *coli* cells (Library Efficiency DH5α competent cells; ThermoFisher Scientific). Psi-mut1-2 was generated from the amplification of psi-mut1 with primers harbouring miR-23 site 2 mutation (Fw-sense- 5′caaaacaatgtgaaacattaaaattaaaaggcat-3′, Rv-sense-5′gccttttaattttaatgtttgccagtgttttg-3′).

The correct nucleotide sequence of amplified products were verified by sequencing of all the generated constructs.

### Protein extraction and Immunoblot analysis

Cells and tissue samples were lysed in RIPA buffer (150 mM NaCl, 50 mM tris-HCl pH8, 1 mM EDTA, 1%NP40, 0.25% sodium deoxycholate, 0.1% SDS), supplemented with protease and phosphatase inhibitor cocktails (Sigma-Aldrich). The determination of protein concentration was measured by colorimetric assay using the pierce BCA protein assay kit.

For the immunoblot analysis, an equal amount of proteins (20–30 µg) was resolved in SDS- polyacrylamide gels (10–12%) and transferred onto PVDF membranes (Amersham). Saturated membranes with 5% non fat dry milk in PBS- Tween (0, 01%) were incubated over-night with specific primary antibodies. The immunoreactive protein bands were detected by incubation with horseradish peroxidase-conjugated secondary goat anti rabbit (Millipore) or goat anti mouse (Sigma-Aldrich) antibodies. The western blot images were acquired on a ImageQuant LAS 4000 (GEHC) and quantified by ImageJ software.

The following antibodies were purchased from Santa Cruz Biotechnology: myogenin (sc-576) cyclin D1 (sc-718), TrxR1 (sc-20147), β-actin (sc-47778), MyHC (sc-20641) and p21(sc-397). GAPDH (MAB 374) antibody was purchased from Millipore.

### RNA extraction and RT-qPCR analysis

Total RNA extraction was performed by using Tri-Reagent (Sigma) according to the manufacturer’s instructions.

For gene expression experiments, the reverse transcription (RT) and qPCR steps were conducted in the same reaction well by using power SYBR green RNA-to-CT 1 step kit (ThermoFisher Scientific), following manufacturer’s guidelines.

Specific primers were used: Myogenin sense-5′gaccctacaggtgcccacaa-3′, antisense-5′acatatcctccaccgtgatgct-3′; Cyclin D1 sense-5′cgtggcctctaagatgaagg-3′, antisense-5′tgttctcatccgcctctggc-3′; p21 sense-5′cggtggaactttgacttcgt-3′, antisense-5′cagggcagaggaagtactgg-3′; Myf5 sense-5′ggctggtcactgcctcatgt-3′, antisense-5′cttgcgtcgatccatggtagt-3′; Pax7 sense-5gccctcagtgagttcgattagc’-3′, antisense-5′tccttcctcatcgtcctctttc-3′; Cyclophilin A sense-5′gtcaaccccaccgtgttctt-3′, antisense-5′ctgctgtctttgggaccttgt-3′.

TaqMan method was employed for microRNA expression analysis. Briefly, 10 ng of total RNA was reverse-transcribed using TaqMan microRNA reverse transcription kit (Applied Biosystems, 4366596) following manufacturer’s instructions. Then 1.3 µl of each miR-specific cDNA was submitted to PCR amplification by using Taqman universal PCR master mix II (Applied Biosystems, 4440044). The following TaqMan microRNA assays were used as probes: hsa-miR-23a (000399), hsa-miR-23b (000400), hsa-miR-206 (000510) and U6 snRNA (001973).

The comparative cycle threshold (ΔΔCt) method was used to analyze the relative expression levels using cyclophilin A or U6 snRNA as internal controls.

### Luciferase reporter assay

C2C12 cells were transfected with psi-wt, psi-mut1 or psimut1-2 constructs by using Lipofectamine 2000 transfection reagent (ThermoFisher Scientific). After 24 hours, differentially transfected cells were detached, adjusted to a concentration of 35.000 cells/ml and reverse-transfected with mimic miR-23a and miR-23b alone or together, or with a synthetic scramble-sequence miRNA. 24 hours after plating, luciferase’s activity was measured by using Dual luciferase reporter assay system protocol (Promega), following manufacturer’s recommendations. The values reported in the graphic represent the ratio between renilla and firefly activities.

### *In silico* prediction for miRNA binding sites

Bioinformatic search for miR-23 a and b binding sites in 3′UTR of TrxR1 was performed by using TargetScanHuman 7.0 (www.targetscan.org).

### Cell growth assay

3 × 10^4^ reverse-transfected cells/well were seeded in a 96-well plate. After 24, 48 and 72 hours, cell viability was measured using Cell titer aqueous assay (Promega) with MTS tetrazolium following manufacturer’s instructions. Four independent experiments were performed in triplicate.

### Cytofluorimetric analysis

C2C12 cells were collected and fixed in EtOH 70%. After washing, samples were incubated with RNAse A (0.5 mg/ml) for 15 min. Propidium Iodide (PI) was then added at the final concentration of 20 µg/ml for 15 min at 37 °C. Cytofluorimetric analysis was performed on three independent experiment by using Facscalibur. *Redox-blotting* Trx-1 redox state in myoblasts during proliferation and differentiation (D1-D5) was analysed as previously described^46–48^ with minor modifications. To determine the proportions of Trx-1 in the reduced and oxidized forms, cells were resuspended in lysis buffer (50 mM Tris/Cl, pH 8.3; 3 mM EDTA; 6 M Guanidine HCl; 0.5% Triton X-100; 1% protease inhibitor) containing 5 mM iodoacetic acid (IAA) at 37 °C for 30 minutes in the dark. Cell lysates were collected and filtered using a protein desalting spin column (Pierce) to remove excess IAA. Eluates were then diluted in 5× native and non-reducing sample buffer and separated by native polyacrylamide gel electrophoresis (6% stacking gel, 15% resolving gel). Gels were electroblotted to polyvinylidene difluoride membrane and probed for Trx-1 using anti-Trx-1 primary antibody (1:2000; Abcam) followed by a horseradish peroxidase-conjugated anti-rabbit secondary antibody. Bands corresponding to Trx-1 were detected using the CCD camera of a FujiFilm LAS-4000 analyser.

### Immunofluorescence analysis

For immunofluorescence analysis, muscle cells were fixed with 4% formaldehyde and permeabilized with 0.2% Triton X-100. To avoid unspecific binding, muscle cells were first blocked with a solution containing 6 ng/ml IgG from goat serum or BSA 5% in PBS. Dilutions of the following primary antibodies were incubated for 1–2 hours at RT in protocols relative to SCs: mouse anti-MyHC (1:200; MF20, MAB447), rabbit anti-TrxR1 (1:100; SC-20147). Specimens were then incubated with Alexa Fluor 488-conjugated anti-mouse IgG (H + L) or Alexa Fluor 594-conjugated anti-mouse IgG (H + L) secondary antibodies (Life Technology, 1:500) for 1 hour at RT. In C2C12 myoblasts and differentiated myotubes the anti-MyHC antibody (sc-20641) (1:300) or rabbit anti-TrxR1 were incubated overnight at 4 °C and the Alexa Fluor 488-conjugated anti-rabbit IgG (H + L) was used as secondary antibody (1:500). Hoechst dye (0.1 mg/ml) or DAPI (0.5 mg/ml) were added for the last 10 min to stain nuclei. Slides were mounted in Mowiol 4–88 reagent (Calbiochem) and images were taken using a Leica inverted microscope. Images (20X) were saved as TIFF files and Photoshop (Adobe) was used for composing panels.

### Fusion Index

To evaluate myogenic differentiation, a fusion index was calculated to indicate myotube fusion. Fluorescent images of random fields were captured with 20X magnification. For each experimental condition, total number of nuclei and number of nuclei incorporated into the myotubes were counted. Fusion index was calculated as the percentage of nuclei incorporated into myotubes (defined as containing at least two nuclei) relative to the total number of nuclei.

### Statistical Analysis

Statistical analysis was performed using GraphPad Prism 5.03 and p values were calculated using the unpaired t test. Statistically significant differences are marked with asterisks in the figures (*p < 0.05; **p < 0.01; ***p < 0.001). All results represent the means ± SEM of at least three independent experiments.

### Ethics statement

Animal experiments were performed according to protocol number 809_2015PR, following the Institutional guidelines of the Fondazione Santa Lucia and the approval of the Ethical Committee.

### Data availability

The datasets generated during and/or analysed during the current study are available from the corresponding author on reasonable request.

## Electronic supplementary material

Supplementary information
