# Supplementary material for: MiR-23-TrxR1 as a novel molecular axis in skeletal muscle differentiation
Source: Sci Rep. 2017 Aug 3;7:7219. doi: 10.1038/s41598-017-07575-0 (PMC5543121; doi:10.1038/s41598-017-07575-0)

MiR-23-TrxR1 as a novel molecular axis in skeletal muscle differentiation

Neri Mercatelli, Simona Fittipaldi, Elisa De Paola, Ivan Dimauro, Maria Paola Paronetto, Malcolm J Jackson, Daniela Caporossi

SUPPLEMENTARY INFORMATIONS

Supplementary Figure 1. TrxR1 is expressed in skeletal muscle tissues and its expression is downregulated during C2C12 muscle differentiation

a) Western blot analysis showing the protein contents of TrxR1 in *gastrocnemius* (G), *extensor digitorius longus* (E), *soleus* (S) and C2C12 myoblasts (M). **b)** Immunofluorescence analysis showing TrxR1 expression during C2C12 muscle differentiation (scale bar: 50µM, magnification: 40X).

Supplementary Figure 2. The oxidative status of Trx1 does not change during C2C12 muscle differentiation.

Representative image of the redox western blot showing the redox status (red: reduced, ox: oxidized) of Trx1 in C2C12 cells myoblasts (GM) treated ($NaAsO_2$ for 1 hours) or not (Ctrl) with $NaAsO_2$ and collected at different time points of differentiation (1, 2, 3 and 5 days).

Supplementary Figure 3. TrxR1 siRNA specificity.

a) western blot showing the reduction of TrxR1 expression after siTrxR1 siRNA N°1, N°2 and N°3 transfection. **b)** RT-qPCR analysis showing the expression levels of pax7 and myf5 mRNA.

Supplementary Figure 4. TrxR1 is necessary to myogenic process.

C2C12 were transfected or not (mock) with TrxR1 siRNA or a scramble siRNA (scr siRNA) and analysed at D1, D2 and D4 from differentiation induction. **a)** Representative image of the western blot analysis showing the protein expression of TrxR1, MyHC, Myogenin and β-actin. The amount of TrxR1, MyHC and Myogenin **(b)** in each sample was calculated by normalizing to β-actin protein levels. Results are represented as mean ± SEM of at least three different experiments. **c)** Representative image (20x magnification) of the immunofluorescence analysis showing MyHC expression at D3 of differentiation. **d)** Fusion index relative to D3.

Supplementary Figure 5. miR-23 a and b overexpression reduces the mRNA levels of TrxR1 and negatively affects C2C12 cellular growth.

C2C12 were transfected with miR-23 a, miR-23b or scr mimic and cultured in GM conditions. **a)** RT-qPCR showing the expression levels of TrxR1 mRNA at 36 and 48 hours after transfections. The value of scr miR samples was arbitrarily set=1. **b)** MTS assay measured (OD) at 24, 48 and 72 hours after transfection.

Supplementary Fig. S1

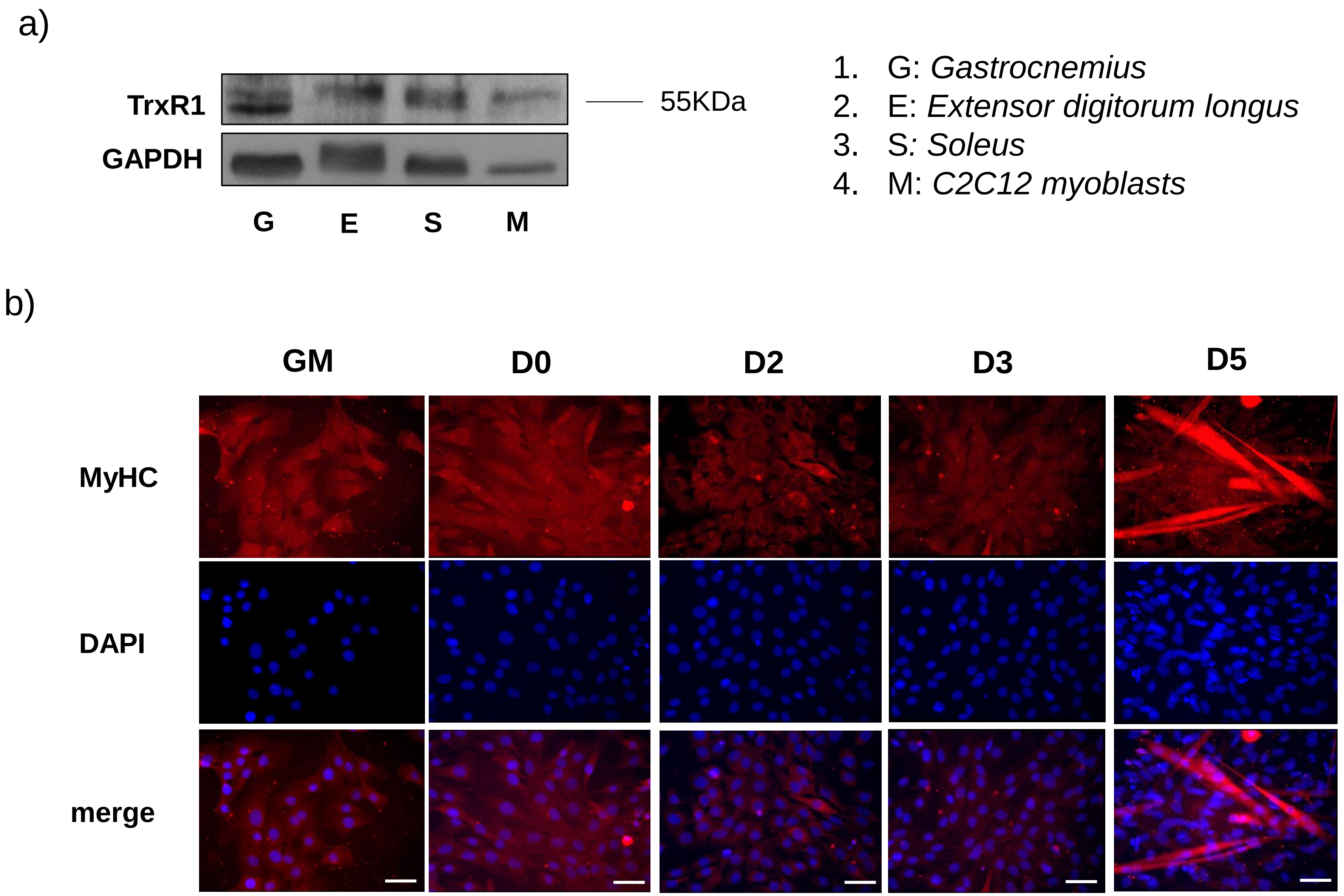

Supplementary Fig. S2

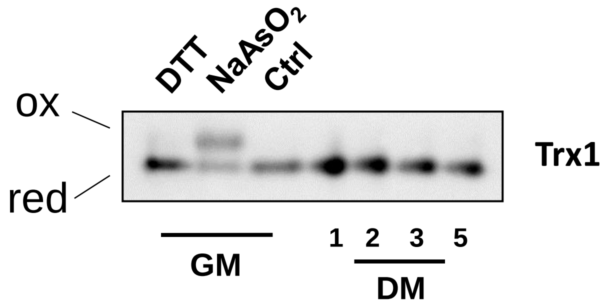

Supplementary Fig. S3

a)

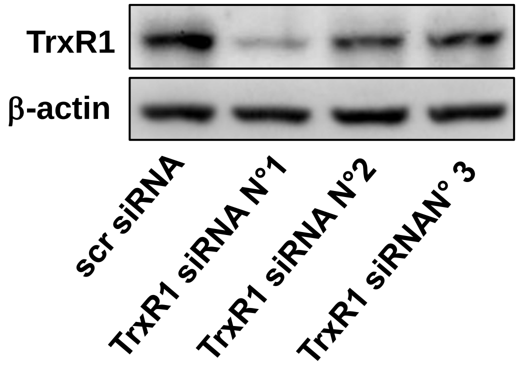

b)

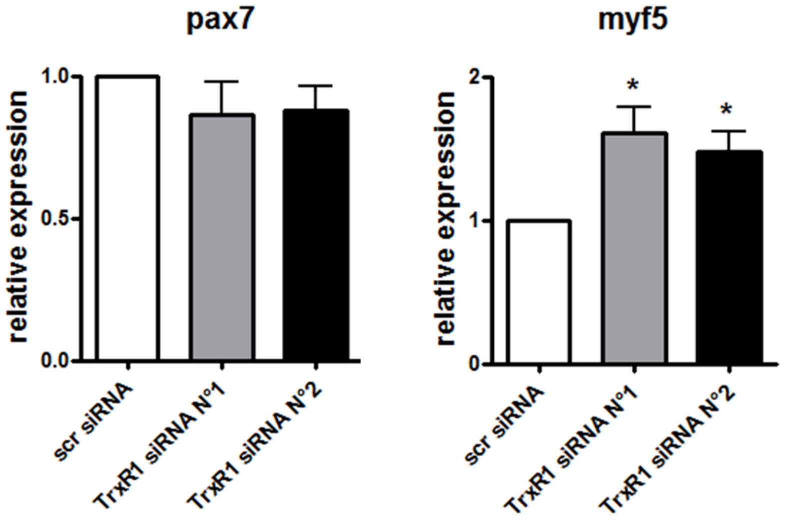

Supplementary Fig. S4

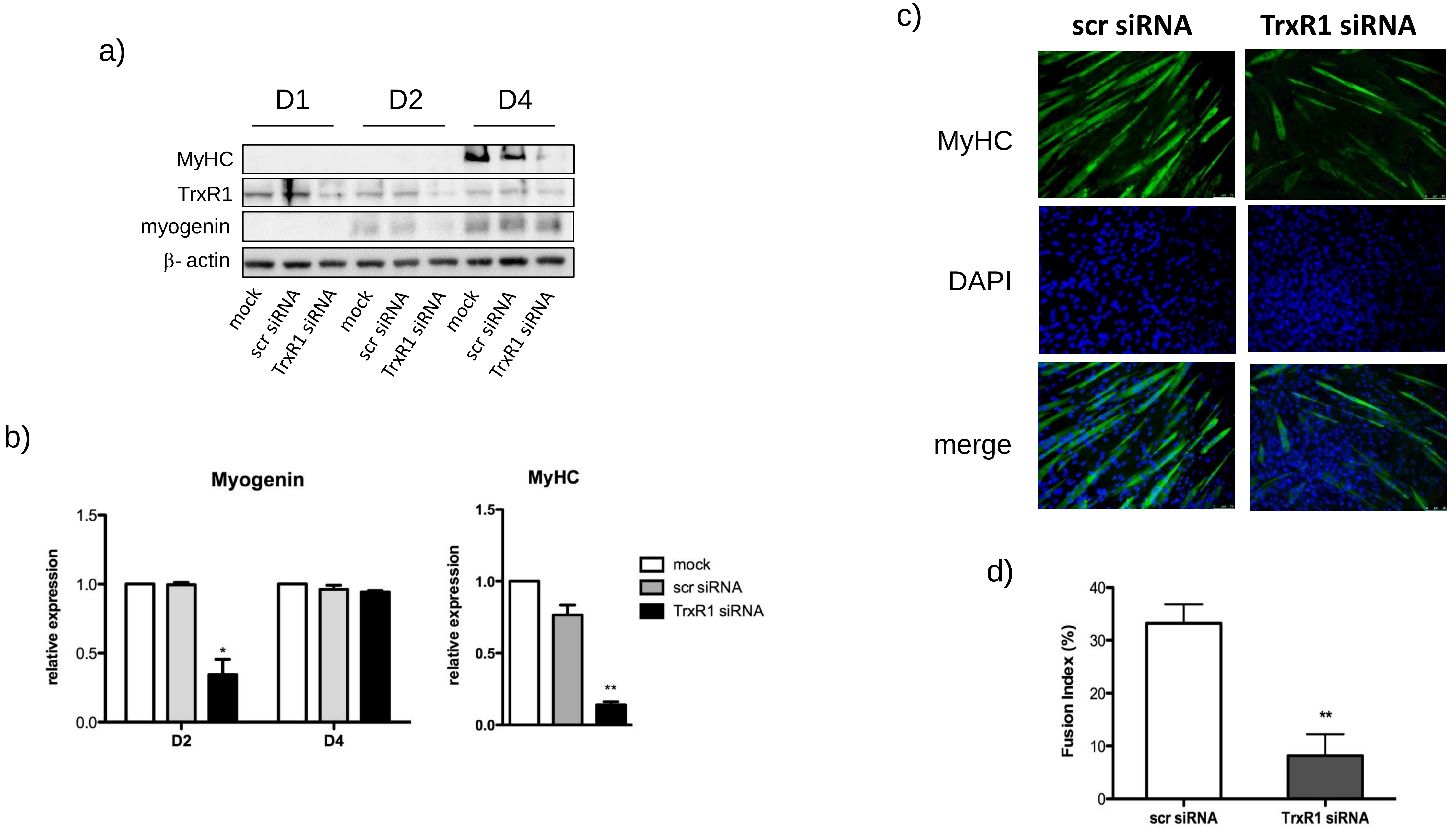

Supplementary Fig. S5

a)

b)

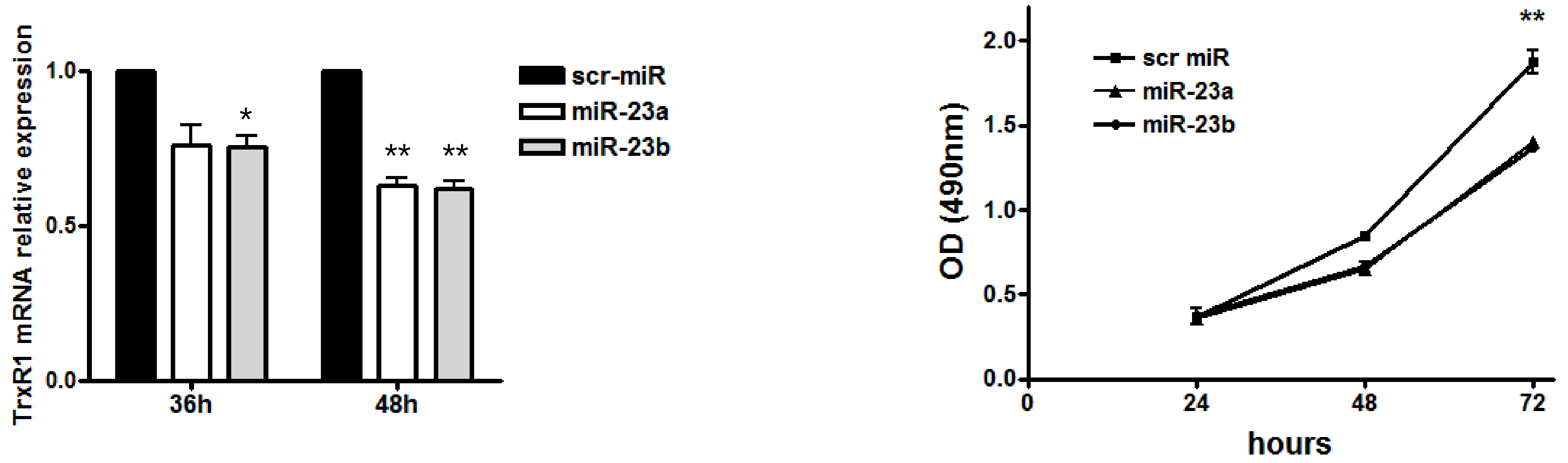

Supplement: Supplementary file 1 — Supplementary information [file 41598_2017_7575_MOESM1_ESM.pdf]
